# Efficacy of Diode Laser Ablation and Low Level Laser Therapy on Healing and Bacterial Load Reduction at Intraoral Biopsy Site

**DOI:** 10.30476/DENTJODS.2021.88696.1354

**Published:** 2022-06

**Authors:** Aayushi Gaur, Manu Dhillon, Nidhi Puri, Upasana Sethi, Shivangi Singh, Seema Ahuja

**Affiliations:** 1 Postgraduate, Dept. of Oral Medicine and Radiology, I.T.S Centre for Dental Studies and Research, Muradnagar, India; 2 Dept. of Oral Medicine and Radiology, Divya Jyoti Dental College, Modinagar, India; 3 Dept. of Oral Medicine and Radiology, I.T.S Centre for Dental Studies and Research, Muradnagar, India

**Keywords:** Diode laser, Ablation, Bacterial load, Healing, Analgesia, Biopsy

## Abstract

**Statement of the Problem:**

Healing complications after the conventional surgical biopsy procedure along with bacterial colonization indicates scope for sophisticated techniques. Amalgamation of photo-disinfection along with healing properties of diode laser with practiced biopsy technique can help in dealing with post biopsy complications.

**Purpose:**

The present study will analyze the possibility of conjugation of conventional surgical biopsy technique with diode laser regarding its superior properties for achieving better healing and analgesia along with sterilization of the biopsy site.

**Materials and Method:**

A randomized control trial was done where punch biopsy procedure was performed for homogenous leukoplakia. Patients were randomly divided into laser group (Test group) and control group. Test group received laser ablation and low level laser therapy (LLLT) on surgical site along with warm saline rinses whereas control group was prescribed with systemic analgesic and antibiotics. Pain on visual analogue scale (VAS), erythema along with the size of defect was evaluated on day 0, 2 and 4. Swabs were collected from the biopsy site and culture was done for evaluation of bacterial load.

**Results:**

Highly statistical significant values indicating laser induced analgesia were obtained after analysis for 2nd and 4th day (*p*= 0.00). Erythema and biopsy defect size evaluation showed significant results for 2nd day (p value 0.023 and 0.004 respectively), which showed absence of erythema and enhancement of healing in test group compared to controls. Statistical significant results were obtained for estimation of bacterial colonization with p value as 0.00, 0.00 for 2nd and 4th day claiming laser supported bacterial disinfection. There was a significant percentage increase on 2nd (*p*= 0.013) and decrease on 4th post-operative day (*p*= 0.022).

**Conclusion:**

The results encourage the conjugation of conventional incisional punch biopsy with low level lasers to avoid systemic intervention for post biopsy complications.

## Introduction

Rapidly advancing oral disease spectra necessitates the call for more sophisticated approach towards diagnosis either as an alternative or as an adjunct to the conventional diagnostic techniques. High incidence and increasing prevalence of oral cancer has attracted the needed attention to the potentially malignant disorders such as leukoplakia, oral sub mucous fibrosis and erythroplakia, and their direct influence on the survival [ 1 ].

Leukoplakia with a known transformation rate as high as 0.6-20 percent, is a well-accepted potentially malignant disorder of oral cavity [ 2 ]. The possibility of histopathological evaluation has helped the treatment modalities evolve around the specific characteristics of diseases and their underlying pathophysiology [ 3 ]. Along with clinical examination, incisional biopsy, as the conventional technique, has been broadly used for diagnosis of oral leukoplakia. The surgical biopsy technique can be considered as a double edge sword for having strengths such as precision, low cost, and favorable healing where on the other hand it has liabilities such as hemorrhage, post procedure discomfort, pain, and secondary complications like infection [ 3 ].

Recently, the incisional biopsies using surgical punches have gained popularity among diagnosticians, regarding its many advantages over scalpel biopsies such as cost effectiveness, low technique sensitivity, and controlled approach [ 3 ]. However, one of the disadvantages of surgical punches is that the defect created by them heals by secondary intention. The absence of sutures encourages the formation of granulation tissue followed by the scar formation. In addition, there are greater possibilities of secondary complications like superadded infections [ 4 ].

In healthy patients, prescription of antibiotics after the intraoral incisional biopsy is debatable; however, because of the poor oral hygiene in such patients due to continuous tobacco consumption and avoidance of oral hygiene habits after biopsy, antibiotics are often prescribed to avoid super infection. Prophylactic prescription of antibiotics were prescribed for “just in case” phenomenon to avoid any chances of secondary complications in most of the patients [ 5 ]. Injudicious prescription of antibiotics has led to antibiotic resistance, which is an alarming global issue. Antibiotic and analgesics are also known to have several side effects like gastric upsets and antibiotic resistances [ 6 ]. This problems demand for an alternative approach for providing better treatment outcome without the use of systemic medications.

Laser has emerged as a latest technique that can be used to overcome the complications of conventional biopsy techniques. The lasers are ranging from low to high intensities and varied wavelengths have many properties such as photo disinfection, analgesia, anti-inflammatory, and bio stimulatory effect, which have made them a boon in dentistry and medicine [ 7 ].

Systemic analgesics can be avoided by application of low level laser therapy (LLLT) due to their documented targeted neuro-pharmacological effect on physiology of neuro-chemicals, which are responsible for pain perception at peripheral level and thereby highly effective in pain reduction [ 8 ]. Furthermore, stimulation of cell regeneration and differentiation, especially for the fibroblast along with their conversion into myofibroblasts encourage the healing process that results in superior quality healed tissue [ 7 - 9 ]. All of these properties justify the exploration of effectiveness of LLLT therapy in post-surgical pain and healing time reduction.

To our best knowledge, there have been no studies, which emphasize on the enhanced healing effect of LLLT on the surgical defect made by surgical punches during oral mucosal biopsies in patients with potentially malignant disorders and possible aversion towards the use of systemic administration of analgesics and antibiotics. The present study was conducted to determine the possibility of amalgamation of conventional incisional punch biopsy with LLLT using diode laser for its superior properties like field disinfection and analgesia to induce healing of surgical site.

## Material and Method

The current study was single center, randomized controlled trial carried out in the Department of Oral Medicine and Radiology and Advance Research Lab at ITS Centre for Studies & Research, Ghaziabad, Uttar Pradesh, India. 

### Ethical statement

The proposal of this *in vivo* clinical trial was reviewed and ethically approved (Protocol No.- ITSCDSR/IIEC/ RP/2018/021) by the Institutional Review Board of the I.T.S Centre for Studies & Research, Ghaziabad, Uttar Pradesh, India. Patients were explained about the trial and written consent was taken before enrolment. 

### Study participants

On the basic of statistical analysis of the pilot study, which was done initially on smaller patient group, sample size comprising of 42 patients from the Department of Oral Medicine and Radiology at I.T.S Centre for Studies & Research, Ghaziabad, Uttar Pradesh, India were taken as study participants (21 patients in each group). All the participants underwent punch biopsy procedure. Group 1 (test group) received laser intervention in the foam of biopsy defect ablation followed by biostimulation whereas group 2 (control group) received antibiotics and analgesics as systemic intervention. The patients were informed regarding the procedure separately and were unrelated to each other to negate the possibility of any psychological bias regarding the perception of pain. The simple sampling method was adopted for this study where each individual was chosen entirely by chance and each member of the population fitting the inclusion criteria has an equal chance, or probability, of being selected for any of the groups. The patient who fitted the inclusion criteria were included in the study randomly in sequence of their arrival i.e. the patients were allotted the group alternatively. The patients were diagnosed based on habit history and chair side examination by three experienced oral medicine and diagnostic specialists were included in the study. 

### Inclusion criteria

The inclusion criteria were defined as (1) male patients, (2) participants having the age of 20 to 60 yrs, who were clinically diagnosed with homogenous leukoplakia in accordance with the definition given by Warnakulasuriya *et al*. [ 2 ], and (3) patients with no systemic disorders like diabetes, hypertension, and heart disease with/ without H/o surgical procedures.

### Exclusion criteria

The exclusion criteria were defined as (1) female patients (in order to maintain the homogeneity in the subject and reducing bias), (2) patients with age less than 20 or more than 60 yrs., (3) patients on any systemic medication, and (4) patients provisionally diagnosed with potentially malignant disorders other than homogenous leukoplakia.

### Biopsy sample collection and preservation

Biopsy site was determined after clinical examination and preoperative bacterial swab was collected from the biopsy site from a circle of diameter 1cm with biopsy site as epicenter using sterile cotton swab on day 0 and on follow up days (2nd and 4th day). The sample was diluted in saline in autoclaved Eppendorf tube and stored immediately at minus 20 degree Celsius temperature. Incisional biopsy was then performed using 6 mm punch under local anesthetic infiltration. Hemostasis was achieved by applying pressure by sterile gauge for about 5-7 minutes. 

### Laser application

Diode laser (BioiLase™, iLase™ dental soft tissue laser, InGAsP Semiconductor diode, wavelength 940 nm, power 1.50W, continuous mode, contact mode with 200 micro meter fiber tip) was used in custom mode to ablate the core biopsy defect for the study group. Further, the LLLT was done to achieve biostimulation with same diode on adjacent mucosa on custom mode (wavelength 940 nm, power 0.6 W, 3 cycles of 10 seconds each) whereas Betadine cotton pack was placed for at least 45minutes for the patients in the control group. 

### Post-operative instructions

Patients of both groups were discharged from the operatory and postoperative instructions were given. Both the groups were advised to do warm saline rinses at least 4 times a day. No prescription for antibiotics or analgesics was given in test group whereas the participants of control group were prescribed both antibiotics and analgesics along with warm saline rinses. Patient of the test group were asked to contact the doctor in case of moderate to severe pain, swelling or any post-operative complications. Patients were advised to follow their regular oral hygiene and were recalled after 2 days for review of the biopsy defect. 

### Size detection

The size of the defect on day zero was standardized by using surgical punch of 6mm diameter for all patients. The size of the defect was evaluated using divider and scale in 2nd and 4th day subsequently. 

### Pain evaluation

Pain was evaluated on 2nd and 4th day using visual analog scale (VAS) where “0” was no pain and “10” was worst imaginable pain. 

### Erythema evaluation

For erythema evaluation, standardized clinical photographs were taken on each visit. The photograph from the previous visit was compared with the photograph from the day of visit by single operator. Erythema was evaluated objectively as present or absent during each visit by single operator to avoid bias. 

### Bacterial load estimation

The bacterial samples were taken out from the refrigeration unit and were allowed to be normalized under room temperature. The sample was prepared at-2 dilution and was inoculated in Petri dishes carrying brain heart infusion (BHI) media using sterile cotton swab. The inoculated media was then allowed to incubate in temperature-regulated incubator where the temperature was set to be 37 degrees for 24 hrs. After the incubation period, the colony forming units were assessed using Simtronics digital colony counter.

### Patients follow up

Patients were then followed up by the operator for two weeks and bacterial swab was repeated on day 2 and 4. Before collection of swab on follow up visits, the defect was first cleaned superficially with normal saline to get rid of any necrotic slough and then the bacterial swab was collected from the 1mm diameter circle with defect as epicenter as earlier.

### Statistical analysis

Data was formulated and then statistically analyzed by using statistical package for social science software 19.0 (SPSS Inc. Chicago, IL, USA) by applying Chi square test, Levene’s test and T-test. p value of less than 0.05 was considered as statistically significant for all the tests.

## Results

A total of 42 patients with a mean age of 39 yrs. (minimum age 18 and maximum age 60) were included in the study in accordance to inclusion and exclusion criteria. After clinical evaluation, patients were divided into 2 groups including the study group; who were exposed to laser ablation, that comprised of 21 patients (Group 1) and the control group constituting 21 patients (Group 2); in which topical beta dine was applied. 

**Figure 1:**
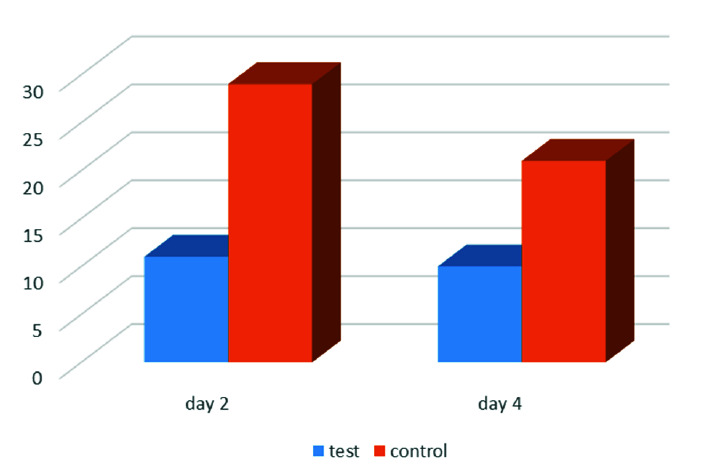
Graphical presentation for the Mean rank of visual analogue scale (VAS) scores in by Mann Whitney test in group 1 (test) and group 2 (control) on day 2 and day 4

Patients were followed up and pain was evaluated on 2nd and 4th day on VAS. The statistical analysis done by Mann Whitney test showed the mean rank as 11 and 29 for study and control group respectively on day 2. Similarly, on day 4, the mean rank for study and control group was 10 and 21, respectively. The *p* Value for VAS scores for both 2nd and 4th day was 0.00 which showed that there was statistically high significance and suggests that group1 had much satisfactory analgesic effect as compared to group 2 (Figure 1).

The erythema evaluation revealed that the biopsy site for the group 1 showed less or no erythema in comparison to the group 2. The statistical analysis using Chi-Square test revealed p value for day 0, 2nd and 4th day as 0.123, 0.023 and 0.133, respectively, which indicated that finding were statistically significant for day 2 only and not for day 0 or day 4.

**Figure 2:**
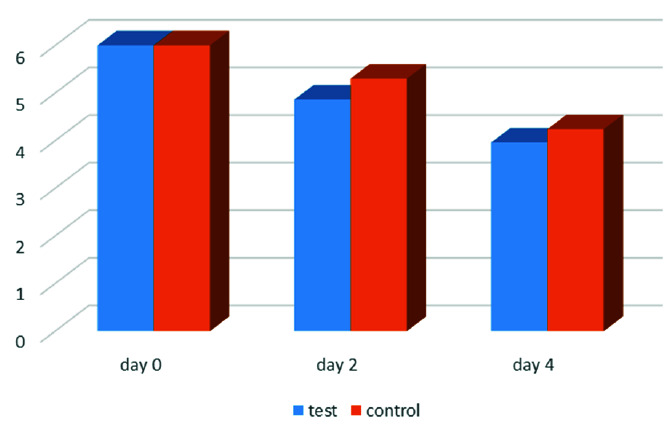
Graphical representation of “mean of size” on day 0, 2nd, 4th in control and test group

**Figure 3:**
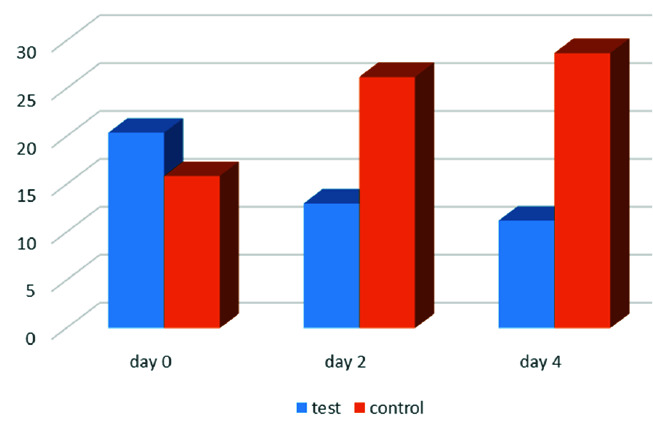
Graphical representation of the mean rank for day 0, 2nd and 4th day by Mann Whitney test for bacterial colonisation for test and control group

**Figure 4:**
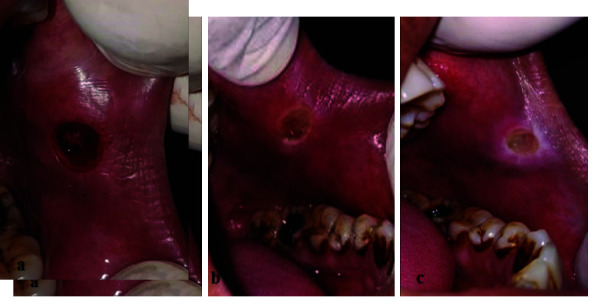
Picture depicting healing in control group (group 2); **a:** day 0, **b:** day 2, **c:** day 4

**Figure 5:**
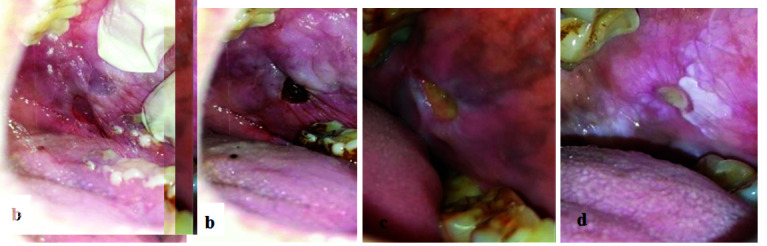
Picture depicting healing in test group (group 1); **a:** day 0, **b:** laser ablation, **c:** day 2, **d:** day 4

Healing after biopsy was evaluated based on change in the size of the surgical defect over the follow up visits on 2nd and 4th day. The biopsy was done with as 6mm punch to maintain the baseline for both the groups. The mean value of size on 2nd and 4th day for group 1 and group 2 are presented in Table 1. The reduction in the size of the lesion was statistically significant on 2nd day only (*p*= 0.004) (Figures 2-5; Table 1).

**Table 1:** Tabular presentation of statistical analysis for changes in size of the surgical biopsy defect in test and control group

Group		Mean	Std.Deviation	*p* Value
Size 0	Test	6.00	.00	-
	Control	6.00	.00	
Size 2	Test	4.88	.415	0.004
	Control	5.30	.364	
Size 4	Test	3.957	.692	0.289
	Control	4.233	.842	

Bacterial counts were evaluated using Colony Counter and obtained values were subjected to Mann Whitney test for statistical analysis as well. Mean rank obtained by the statistical test for day 0, 2nd, and 4th day for test group were 20.38, 13.00 and 11.21, respectively.

For control group, the mean rank for day 0, 2nd and 4th day were 15.87, 26.20, and 28.70, respectively. The mean ranks of control and test group clearly suggested that the bacterial colonization for control group was significantly higher as compared to the test (laser ablated) group (Figure 3). The *p* Value for 0, 2^nd^ and 4th day were 0.203, 0.00 and 0.00, respectively. The obtained data showed that the bacterial counts increased on day 2 and decreased on day 4 for both the groups. 

To analyze the percentage increase on day 2 and percentage decrease on day 4 as compared to day 2, independent t test was applied to the obtained data. The statistical analysis showed that the mean value of percentage increased in bacterial load on day 2 with day 0 as baseline for group 1 and group2 on day 577.38 and 1072.43, respectively. These values signified that although the bacterial load has increased but the percentage increased in almost 50 percent less in LLLT group (group1) as compared to non-laser group (group2) on day 2. Similarly, the mean of percentage decrease in bacterial load on day 4 with day 2 as base line, in group1 and group 2 was -28.913 and -50.194, respectively (Table 2). The significantly higher percentage difference in decrease on bacterial load on day 4 also substantiated the efficacy of LLLT for its antibacterial effect. The p value for difference in percentage increase on day 2 was 0.013 and value for percentage decrease on day 4 was 0.022, which were statistically significant (Table 2).

**Table 2:** Tabular presentation statistical analysis of percentage increase and decrease in bacterial load on day 0, 2 and 4 (Bacp 02: bacterial percentage of day 2 with day 0 as baseline, Bacp 24: bacterial percentage of day 4 with day 2 as baseline)

Group		Mean	Standard deviation	*p* Value
Bacp 02	Test	577.38	491.629	0.013
	Control	1072.43	635.941	
Bacp 24	Test	-50.194	25.2488	0.022
	Control	-28.913	27.3384	

## Discussion

There is an evolving hue of oral lesions along with pounding incidence and prevalence around the world [ 2 ]. India is one of the top ten countries having an exponential rise in premalignant disorders like leukoplakia and oral sub mucous fibrosis [ 10 - 11 ]. In 1879, Ernest coined the term biopsy and since then, it has been accepted as the gold standard diagnostic procedure for the definitive diagnosis of varied range of suspected oral condition and lesions [ 12 ]. Conventional scalpels provide ease of use, accuracy and minimal damage to the surrounding tissues but along with that, there are high chances of hemorrhage due to chances of vascular injury, during the procedure [ 3 ]. Most of the patients are even apprehensive and reluctant for the sutures used for hemostasis and faster healing.

When a laser is absorbed, it elevates the temperature and produces photochemical effects depending on the water content of the tissues [ 10 ]. When a temperature of 100°C is reached, it causes the denaturation of the protein in the region of interest. The vaporization of the water within the tissue occurs along with protein denaturation [ 10 ]. Destruction of the cytotoxic sub-epithelial lymphocytes in the diseased epithelium reduces the chances of secondary infection and the charred layer acts as dressing which provides both hemostasis for maintenance of homeostasis [ 10 ].

The improved understanding of healing process and tissue interactions has led to a new era of application of more sophisticated approach rather than repertoire available conventional techniques. Statistical analysis of the data of present study showed that healing in the laser-irradiated tissue was better as compared to the control group on day 2 with minima enhancement seen on 4th day. D'Arcangelo *et al*. [ 11 ] in his experiment stated that the healing in surgical defect made by conventional scalpel has superior results as compared to diode laser. Thermal damage, higher inflammatory infiltrate along with slow organization due of cells during initial phases of wound healing were held responsible for delayed healing after laser irradiation. In contrast, Ghalayani *et al*. [ 12 ] in his study to determine the effect of diode laser irradiation on healing stated that there was an evidence of enhanced healing along with generation of iNOS giving histological as well as biochemical proof of the results similar to present study. The use of appropriate parameter like wavelength, frequency, and power are important key players to dominate the healing aspects [ 12 - 13 ]. 

Histological analysis of the surgical intervention of lasers has shown significant difference between the inflammatory infiltrate, which can be the reason for erythema and pain. Efficiency of enhancing the tissue healing can be attributed to its literature-supported effect on fibroblast and vascular tissue proliferation, migration and differentiation, which is accompanied by enhanced epithelial cell division [ 14 ]. Saygun *et al*. [ 9 ] has reported in his study conducted on animal models that the basic fibroblast growth protein, which is a multifunctional polypeptide responsible for the differentiation and proliferation along with the transformation of fibroblast into myofibroblast responsible for the wound contracture, is also stimulated by low level laser irradiation. These entire factors are responsible for establishing the environment for the advancement of proliferative phase into the healing phase. Similar results are observed in our study with higher healing rate in the laser group. Along with the ability to seal off the blood vessels, the ablation not only provides biological dressings but also minimal edema and achievement of homeostasis. This can be one of the reasons for the enhancement of healing in laser group [ 10 ].

Semih ozbayark *et al*. [ 15 ] in his study observed that scalpel wound had better healing index and proposed that laser ablation has delayed the healing and might also caused muscle fasciculation which is in contrast to our study. 

There was a statistical significant increase in the colony forming units (CFU) in both groups in pre- and post-surgical bacterial load in the present study, which could be attributed to the hypothesis that the oral hygiene of the patients must have been compromised concerning their fear of pain and discomfort. Intra-group comparison although showed that mean increase in bacterial load in the control group was higher as compared to the test group. In addition, the mean decrease in CFU on day 4 was also higher in the study group, which further empowers the efficacy of LLLT for the sterilization of the surgical field. The obtained results of present study were in accordance to the results of study done by Gokhale *et al*. [ 16 ] who performed comparative experiment using diode laser of 980nm at 2.5W and comparing with other high power lasers substantiated the superiority of LLLT. The mentioned study stated that the thermo-coagulation of the blood vessels, which resulted in formation of biostatic plug, made due to the charring, helped in prevention of infection over the conventional techniques [ 16 ]. 

Deshmukh *et al*. [ 17 ] stated that laser treatment had significant lower numbers of CFU and attributed it to the absorption of diode wavelength by pigmented bacteria leading to denaturation of protein and vaporization of water, which ultimately resulted in lysis of the cell wall and antimicrobial effect. Laser irradiation is also responsible for the production of reactive oxygen and nitrogen species, which causes further damage to the mitochondria and DNA culmination that, leads to bactericidal effect. Thus, photo stimulated disinfection by LLLT, as targeted therapeutic dose delivery is highly successful and effective as compared to the prescription of biochemical antibiotics and analgesics after scalpel biopsy [ 17 ]. LLLT is biological safe therapeutic modality with no systemic or local side effects observed in the present study and none has been reported in the literature.

Combination of laser therapy along with the conventional surgical and non-surgical methods for reduction of bacterial load has been recently marked based on bactericidal properties of the LLLT. Both low percentage increase on day 2 and high percentage reduction on day 4 in test group compared to the control advocate the use of diode laser after biopsy for reduction of bacterial load.

Along with antibacterial and healing properties, one of the highlights of the present study was the analgesic property of diode laser. Highly statistical significant difference was observed in the VAS scores of test and control groups. 

Analgesic effect may be attributed to the stabilization of the nerve cells and increased accumulation of ATP in neuronal membrane, which affects the pain transmission negatively. LLLT induced stabilization of the lipid bilayers in accordance to the nerve membrane proteins has also been reported [ 14 ]. Alan *et al*. [ 8 ] stated that LLLT has significant neuro-pharmacological effect on synthesis, release, and metabolism of neurochemicals responsible for pain transmission at central and peripheral levels like serotonin, acetylcholine, histamine, and prostaglandin along induced decreased activity of bradykinin on C fibers. Suter *et al*. [ 19 ] in their systemic review stated that LLLT not only reduce the inflammatory processes after surgical procedure similar to systemically given NSAIDs (non-steroidal anti-inflammatory drugs) but also promote nerve regeneration. 

Women experience greater clinical pain, suffer greater pain-related distress and show heightened sensitivity to experimentally induced pain compared with men [ 20 ]. Cycling gonadal hormones along with emotional response to pain have substantial impact on pain perception and analgesic response [ 20 ]. In light of mentioned literature supported facts, inclusion of single gender (males) was preferred for standardizing the study sample.

There are certain limitations of the study like there is no standardized scale for the estimation and evaluation of erythema. The subjective nature of the clinical evaluation makes it questionable. The study can be done on larger study sample to substantiate the results for applicability in general masses. Similar studies in such patients with larger sample size are warranted.

## Conclusion

The results of the present study support the possibility of integration of the diode laser to surgical biopsy technique. The assembly of these two can help not only in enhancing the healing of the surgical defect but also in minimizing the systemic load for antibiotics and analgesics. The encouraging results of test group concluding significant reduction in pain scores as well as bacterial load substantiate the literature in support of laser disinfection and analgesia

## Conflict of Interest

Authors have declared that no competing interests exist.
